# Hints Regarding Children

**Published:** 1892-07

**Authors:** 


### PRACTICAL HINTS REGARDING CHILDREN.

Always teach a nurse that a child cannot swallow as long as the spoon is between the teeth; that it is advisable to depress the tongue a brief moment, and withdraw the spoon at once; and that now and then a momentary compression of the nose is a good adjuvant.

The taste of quinine is disguised by coffee, chocolate and "elixir simplex."

Powders must be thoroughly moistened; unless they be so, the powder adhering to the fauces is apt to produce vomiting.

Inunctions require a clean surface, and are best made where the epidermis is thin, and the net of lymph-ducts very extensive, as on the inner aspect of the forearm and the thigh.

Babies, after having taken opiates for some time, demand larger, and sometimes quite large, doses to yield a sufficient effect.

Febrifuges and cardiac tonics, such as quinine, antipyrine, digitalis, strophanthus, sparteine, convallaria, etc., are tolerated and demanded by infants and children in larger doses than the ages of the patients would appear to justify.

Mercurials affect the gums very much less in young than in advanced age.

The rectum of the young is straight, the sacrum but little concave, the sphincter ani feeble, and self-control is developed but gradually; for these reasons, rectal injection is allowed to flow out or is vehemently expelled. Therefore one which is expected to be retained must not irritate. The blandest and mildest is a solution of six or seven parts of chloride of sodium in a thousand parts of water, which serves as a good vehicle for medicine unless incompatible with the latter. The injection must be made while the child is lying on its side (preferably the left side), not on the belly over the lap of the nurse, for in this position the space inside the narrow infantile pelvis is reduced to almost nothing.

In many cases of intense intestinal catarrh, large and hot (104° to 108° F.) enemata will relieve the irritability of the bowels and contribute to recovery. They must be repeated several times daily. When there are many stools, and these complicated with tenesmus, an injection, tepid or hot must or may be made after every defecation, and will speedily relieve the tenesmus.—*The Nurse.*

